# Shaggy Photoreceptors with Subfoveal Fluid Associated with a Distant Choroidal Melanoma

**DOI:** 10.1155/2015/187542

**Published:** 2015-02-02

**Authors:** Ann Q. Tran, James A. Eadie, Michael M. Altaweel

**Affiliations:** ^1^Department of Ophthalmology and Visual Sciences, University of Wisconsin-Madison, Madison, WI 53705, USA; ^2^Retina Vitreous Consultants, Pittsburgh, PA 15213, USA

## Abstract

*Purpose*. To describe the enhanced depth imaging optical coherence tomography (EDI-OCT) findings in a patient with an extra macula choroidal melanoma before and after treatment. *Methods*. Observational case report. *Results*. A 45 year-old Caucasian male patient was referred to retina clinic for management of choroidal melanoma. Examination revealed a nasal choroidal melanoma while EDI-OCT illustrated subfoveal fluid pocket with elongated shaggy photoreceptors distant and separate from the tumor. The patient was treated with plaque brachytherapy and intravitreal bevacizumab. One week after plaque removal, there was a dramatic reduction in the shaggy photoreceptors. *Conclusion*. Choroidal melanomas have effects that are not localized to the area of the tumor. This loculated pocket of subretinal fluid and coinciding changes to photoreceptor morphology may be related to global changes in choroidal function or release of tumor related cytokines.

## 1. Introduction

The term “shaggy photoreceptors” has been used to describe distinct morphological changes, specifically swelling and elongation of the outer retina as seen on enhanced depth imaging-optical coherence tomography (EDI-OCT). This finding was first described in central serous chorioretinopathy in 2008 [1] and in sympathetic ophthalmia in 2011 [2]. It was later appreciated in choroidal melanomas and choroidal metastases [3, 4] and has been shown to be a feature that distinguishes small melanomas from choroidal nevi [5]. We report a case of an extra macular choroidal melanoma producing a distant subfoveal pocket of fluid with shaggy photoreceptors. The appearance of the outer retina improved following radioactive plaque therapy and intravitreal bevacizumab injection.

## 2. Case Presentation

**Figure 1:**
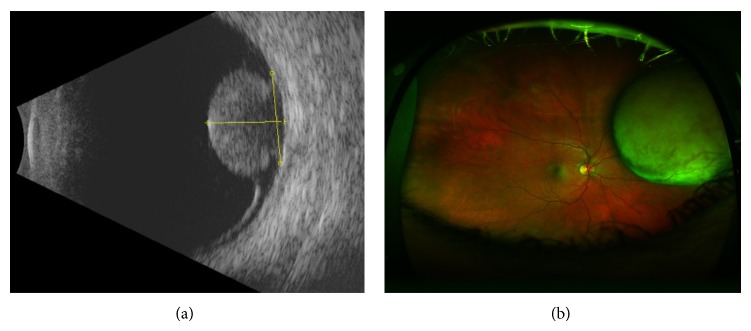
Appearance of the nasal choroidal melanoma (a) and B-scan ultrasonography with some vascularity and low reflectivity but no extrascleral extension (b).

**Figure 2:**
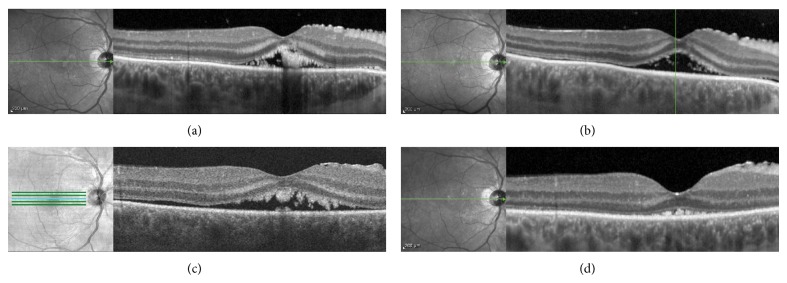
Optical coherence tomography scans of the fovea before (a) and one week after treatment showing reduced volume of shaggy photoreceptors (b). At six weeks, there is recurrent subretinal fluid with presence of shaggy photoreceptors (c) which again resolved four months later (d).

A 45 year-old Caucasian man presented with a one-month history of painless decrease in vision, photopsia, and metamorphopsia. The patient's best-corrected visual acuity was 20/300 OD and 20/20 OS. On dilated exam, he was found to have a medium sized nasal choroidal melanoma and large inferior serous retinal detachment in his right eye (Figure 1(a)). The patient's macula was remarkable for subretinal fluid and striae that did not communicate with the serous retinal detachment inferiorly. B-scan ultrasonography demonstrated a dome shaped choroidal lesion with low internal reflectivity (Figure 1(b)). Tumor base measurements were 9.37 × 13.63 mm with a height of 7.90 mm without extrascleral extension. EDI-OCT of the macula confirmed the presence of an isolated pocket of subfoveal fluid (Spectralis OCT (Heidelberg Engineering GmbH, Heidelberg, Germany); Figure 2(a)). The outer retina was remarkable for shaggy photoreceptors, subretinal lipofuscin precipitates, and a hyperreflective outer plexiform layer.

Three weeks after diagnosis, an 18 mm radioactive plaque was placed on the right eye and was removed 5 days later. Intravitreal bevacizumab (Avastin; Genentech, San Francisco, CA) was given at the time of plaque placement. At the one-week postoperative office visit following plaque removal, best-corrected visual acuity had improved to 20/125 OD. EDI-OCT of the macula showed persistent subfoveal fluid, resolution of subretinal lipofuscin precipitates, and dramatic volume reduction in shaggy photoreceptors (Figure 2(b)). At the six-week followup, his visual acuity remained stable at 20/125 OD. EDI-OCT showed a stable volume of subretinal fluid with potential recurrence of the shaggy photoreceptors in the outer retina (Figure 2(c)). The patient opted to continue with observation versus a repeat intravitreal injection of bevacizumab. At the four-month followup, his best corrected visual acuity was 20/150 OD. Tumor height decreased to 6.05 mm. EDI-OCT demonstrated resolution of subretinal fluid and compressed shaggy photoreceptors (Figure 2(d)).

## 3. Discussion

We report a case of a peripheral nasal choroidal melanoma not involving the macula that produced distant subfoveal fluid and shaggy photoreceptors demonstrated by EDI-OCT. Shaggy photoreceptors appear elongated and irregular. It has been hypothesized that morphological change is due to an incompletely understood effect that the subretinal fluid has on the photoreceptors. This feature was initially described in central serous chorioretinopathy, where the mean length of photoreceptor outer segments measuring 50 *µ*m in the area of subretinal fluid versus the norm of 30 *µ*m in unaffected eyes [6]. More recently, shaggy photoreceptors have been observed in the subretinal fluid overlaying both choroidal melanomas and choroidal metastases [3–5]. They were not observed in eyes with choroidal nevus and overlying subretinal fluid, providing an important feature that assists in distinguishing small melanoma from nevus.

In this case, the subretinal fluid and the shaggy photoreceptors are observed in a localized pocket under the fovea and distant from the tumor itself. The etiology of this fluid is unclear although its presence is potentially indicative of some combination of changes to choroidal permeability, changes to retinal pigment epithelial function, and changes to circulating cytokines related to or produced by the tumor. As there was a serous retinal detachment adjacent to the choroidal melanoma, the subretinal fluid may be explained by tracking from the tumor itself. During I-125 plaque placement, intravitreal bevacizumab was administered, resulting in resolution of the subretinal fluid and improvement in the appearance of the photoreceptors at the two-week postoperative assessment. After four months the shaggy photoreceptors were again present, in association with subfoveal fluid.

The reduction in volume of the shaggy photoreceptors may be related either to the effect of intravitreal bevacizumab administration or to the brachytherapy. Both treatments can result in reduction of subretinal fluid associated with melanoma, and the resolution of the subretinal fluid may be correlated with normalization of photoreceptor appearance. There appears to be a spectrum of photoreceptor changes in association with subretinal fluid. The acute presence of subretinal fluid in this setting results in photoreceptor cellular edema which leads to the appearance of shaggy photoreceptors. When the subretinal fluid is chronically present, the photoreceptor layer atrophies. The loss of the this layer evident on spectral domain OCT, and it has been thought that the shaggy photoreceptors became more granulated and eventually shed [3, 4].

Improvement of shaggy photoreceptors has been reported in patients treated for choroidal metastases [4] and has been associated with resolution of subretinal fluid. This case of choroidal melanoma with subfoveal fluid distant from the lesion demonstrated shaggy photoreceptors that were no longer evident when the subretinal fluid was eliminated and recurred when subretinal fluid reaccumulated. This case adds to the finding that choroidal melanoma with overlying subretinal fluid may be associated with shaggy photoreceptors. Such irregular elongated photoreceptors can be found in distant pockets of subretinal fluid associated with the melanoma. This has not been described with choroidal nevus and therefore serves as a distinguishing factor in suspicious cases.
